# An unusual coincidence of giant cervical leiomyoma and incidental ovarian granulosa cell tumor: A case report

**DOI:** 10.1097/MD.0000000000034387

**Published:** 2023-07-28

**Authors:** Krzysztof Kotowski, Paulina Małyszczak, Magdalena Towarek, Arkadiusz Jagasyk, Marek Murawski, Rafał Sozański

**Affiliations:** a Wroclaw Comprehensive Cancer Center, Wroclaw, Poland; b Lower Silesian Specialist Hospital, Wroclaw, Poland; c Medical University Hospital, Wroclaw, Poland; d First Department of Obstetrics and Gynecology, Wroclaw Medical University, Wroclaw, Poland.

**Keywords:** cervical, CGT, coincidence, leiomyoma, ovarian, tumor

## Abstract

Leiomyomas are the most common benign tumors of smooth muscle origin in women. They are most frequently found in the submucosal tissue of the uterine corpus; however, they also occur in other areas of the uterus, including the cervix. Their size usually varies between 0.5 to 1.0 cm; however, they can reach great dimensions. A strong correlation between the onset and growth of leiomyomas and estrogen levels was observed. Granulosa cell tumor (GCT) is an infrequent sex cord-stromal ovarian neoplasm. Despite their malignancy, GCTs have a good long-term prognosis. In this study, we present a unique case of coincidence of 2 tumors: leiomyoma of rare location (cervix uteri) and extraordinary size (9, 04 cm diameter) with an adult granulosa cell tumor.

**Patient concerns:**

A 67-year-old Caucasian woman was transported from an emergency ward to a gynecological surgery department due to a massive vaginal hemorrhage.

**Diagnoses:**

Preliminary examination showed a presence of an enormous uteri cervix tumor.

**Interventions:**

Initially, the patient underwent physical and ultrasound examinations. To prevent further bleeding, an urgent surgery (hysterectomy) with bilateral salpingo-oophorectomy was performed.

**Outcome:**

Postoperative histopathological examination revealed a cervical leiomyoma and the incidental occurrence of an adult GCT in the right ovary.

**Lessons:**

This case shares an interesting coincidence between a rare variant of leiomyoma and GCT. The study suggests that the potential reason for this can be estrogen secreted by the GCT, which causes the enormous size of the patient’s cervical leiomyoma and the severe vaginal bleeding. Therefore, we advise it is important in abnormal cases to search for other hidden explanations, as in cases of GCT.

## 1. Introduction

### 1.1. Leiomyoma

Leiomyomas are the most common benign tumors among women. It is estimated that approximately 25% of all women of reproductive age present with leiomyomas. The prevalence rapidly increases after menopause, reaching 70%.^[1]^

Leiomyoma initiation and progression strongly correlate with estrogen levels in the systemic circulation. Due to this phenomenon, involution after menopause or ovary removal and an increase in volume during pregnancy may occur.^[2]^

In most cases, leiomyomas are asymptomatic. The most essential symptoms are pathological hemorrhage, polyuria, pain localized in the lower pelvis, and spontaneous abortion.^[3]^

Leiomyomas are well-bordered round tumors. Their size can vary from a few millimeters to enormous sizes, sometimes reaching more than 5.2 kg.^[4]^ Their classification is based on their location in the uterus. Therefore, they are broadly divided into 5 groups: submucosal, subserosal, intramural, cervical, and intraligamental leiomyomas. The last 2 are rare types of leiomyomas. Histopathological classification also divides leiomyomas into other unique subtypes such as *leiomyoma cellulare* or leiomyomas with increased mitotic index values.^[5]^

Notably, cervical leiomyomas are usually smaller in size and measure around 0.5 to 1.0 cm.^[5]^ Nevertheless, the cervical type of leiomyoma commonly occurs in younger women around 30 years of age.^[6]^

The most crucial step in clinical diagnosis is the exclusion of leiomyosarcoma, which is a malignant tumor. In histopathological differentiation exams, the following elements are analyzed: number of mitoses, occurrence of pathological mitosis, polymorphism of cells and nuclei, and presence of necrosis in the tumor area. This microscopic analysis is crucial for patient management due to the different prognoses and treatments of these 2 diseases.^[5,7]^

State-of-the-art treatment is mainly based on surgical uterus removal or hysterectomy with bilateral salpingo-oophorectomy.^[8]^

### 1.2. Granulosa cell tumor

Granulosa cell tumor (GCT) is a rare ovarian cancer, constituting approximately 2% to 5% of all ovarian tumor cases. However, it is simultaneously the most common type of neoplasm derived from the stromal sex cord of the ovary.^[9]^ GCT is classified as a potentially malignant tumor and is mostly diagnosed after menopause as a 1-sided tumor.^[10]^

The vast majority of adult GCTs actively produce estrogen. <30% of GCTs are estrogen-unproductive.^[11]^ Intriguingly, rare cases of GCT-secreting androgens have also been reported.^[12]^ Alterations in hormone levels are crucial because elevated estrogen levels favor the occurrence of a plethora of symptoms such as pathological vaginal bleeding and precocious puberty.^[13]^ Moreover, it forces the occurrence of endometrial hyperplasia, abdominal pain, or even endometrial and breast cancers.^[14]^

Histologically, GCTs are divided into 2 subtypes: juvenile and adult. The less common juvenile GCT occurs in 5% of cases, whereas adult GCTs account for approximately 95% of all GCT cases.^[15]^

The survival rate of patients with GCT is generally high because most patients are diagnosed at an early stage.^[13]^ Unfortunately, particular groups of patients are identified as a high-risk cohort, indicated by a high mitotic index, large tumor size, or rupture. Surgery is an initial, generally sufficient treatment in terms of GCT, whereas adjuvant chemotherapy or radiotherapy is also considered in high-risk patients. The most frequent chemotherapy combinations are bleomycin, etoposide, and cisplatin. Fortunately, relapses or metastases occur in less than 1 in 4 patients.^[16,17]^

## 2. Case presentation

### 2.1. Patient description

**Figure 1.**
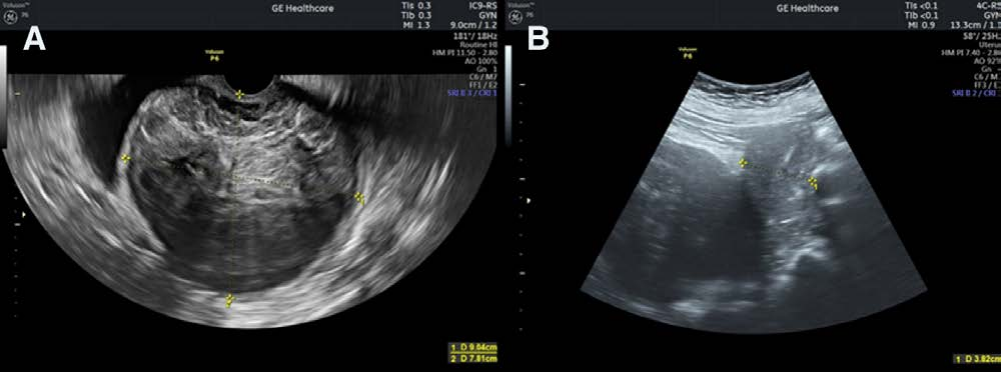
Ultrasonography examination images. (A) Cervical tumor (obtained dimensions - 9.04 cm × 7.81 cm). Intravaginal examination was performed using a Wideband Microconvex Endocavitary Probe (GE Healthcare). (B). The corpus uteri presented with normal dimensions and echogenicity. Peri-abdomen examination was performed using a Wideband Convex Probe (GE Healthcare). Ultrasonography imaging was performed using the Voluson P6 Performance System (GE Healthcare).

An ambulance transported a 67-year-old Caucasian woman from a nearby emergency ward to the gynecological surgery department after a massive vaginal hemorrhage incident. The patient was fully conscious and in an acceptable condition. Abdominal and intravaginal ultrasonography examinations were performed immediately after admission. Examination revealed a uterine tumor with a heterogeneous echo structure. The estimated tumor size was approximately 9 cm, which moved the uterine corpus forward owing to its bulky volume (Fig. 1).

During the primary interview, the patient reported moderate, non-regularly appearing episodes of urine incontinence, stool constipation, and pain in the sacral back area. The patient did not report any pathological vaginal hemorrhage before the incident.

Further interviews provided more detailed information about the patients medical history. Two years prior to the incident, the patient was diagnosed with indolent splenic marginal zone lymphoma. Bone marrow biopsy revealed infiltration of small B-lymphoid cells, constituting 10% to 50% of the examined cells. Immunophenotyping revealed positive staining for CD20, CD79a, and PAX5 in B-lymphoid cells. Furthermore, computer tomography scan performed later showed significant enlargement of the patient’s spleen, which was consistent with previous expectations. The patient reported that after the diagnosis, the patient did not agree with the suggested chemotherapy treatment. The patient did not have any family member’s oncological history.

The patient was pregnant 6 times and gave birth to 6 children. In terms of chronic diseases, the patient reported being under treatment for hypertension with bisoprolol and perindopril. At the time of admission, the only surgical procedure the patient had undergone was laparoscopic cholecystectomy 3 years previously.

Finally, after consideration of all the obtained information, the patient was qualified for surgery: hysterectomy with bilateral salpingo-oophorectomy.

### 2.2. Surgical procedures

**Figure 2.**
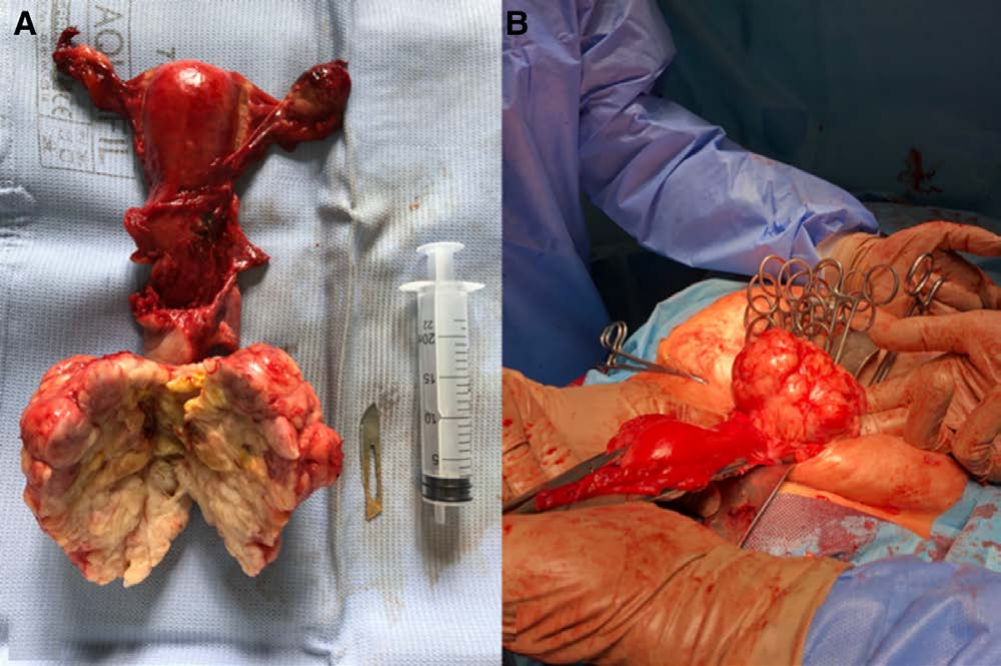
Intra-surgery images: (A) post-excision macroscopic image. (B) Intraoperation image of the tumor during its removal. In the right part of picture A, a surgical scalpel and 20 mL volume syringe is presented to illustrate the dimensions of the removed cervix tumor.

Surgery was performed under general anesthesia. We opened the skin, subcutaneous tissue, and muscles with a longitudinal midline lower abdominal incision and subsequently, after a successful hemostasis, we opened the peritoneal cavity using an electric knife. Next, we visualized the vagina, uterus, fallopian tubes, and ovaries. The cervix of the uterus was altered significantly by the presence of an enormous spherical tumor (Fig. 2A). Macroscopically, the corpus of the uterus and vagina remained unchanged. Notably, at first sight, none of the ovaries presented with evident macroscopic alterations in the post-excision examination. However, the right ovary has attracted our attention and interest. After knot hemostasis and section of round uterus ligaments, suspensory ligaments of ovaries, uterosacral ligaments, and uterine arteries, we removed the uterus with the fallopian tubes and ovaries integrally (Fig. 2B). Subsequently, the surgeon closed the vagina using single knots sutures. Following this, the fascia, muscles, subcutaneous tissue, and skin was closed with stitches. The patient’s overall condition after the procedure was satisfactory. The excised material was subjected to histopathological examination.

### 2.3. Histopathological examination

Macroscopic examination revealed the following dimensions for the uterus: 12 × 4 × 4 cm, with both fallopian tubes and ovaries. The uterine cervix was 8.5 cm long with an uneven border and a huge (9.04 cm diameter) encapsulated polycystic tumor. The cross-section uncovered that the tumor had a whitish and slick structure with extensive areas of necrosis. The uterine muscular layer was 1.8 cm and did not present any macroscopic disease-specific findings. The left ovary was 2 × 1.5 × 1 cm, while the right ovary was relatively larger (3 × 2.5 × 1.5 cm).

Histopathological examination revealed the presence of a leiomyoma tumor of the uterine cervix, showing characteristic co-expression of vimentin, SMA, and desmin. Intriguingly, during a standard examination of all of the surgically removed organs, an adult GCT of the ovary was discovered in the right ovary. Diagnosed GCT presented a specific immunohistochemistry reaction profile – positive vimentin and CA125 with negative results for cytokeratin 7 and cytokeratin 20. To examine the proliferation rate, and thus its malignant potential, the pathologists performed immunohistochemistry staining for a Ki-67. Nearly 5% of the cells were Ki-67-positive.

## 3. Discussion

### 3.1. Leiomyoma

Uterine fibroids are the most common benign neoplasms affecting women, occurring in 70% of the population by the onset of menopause.^[1,18]^ Cervical fibroids are of uncommon localization, and therefore, are classified as a subtype 8, according to the The International Federation of Gynecology and Obstetrics leiomyoma subclassification system.^[8]^ Intense vaginal hemorrhage, presented by our patient, is the most common symptom of uterine leiomyoma.^[7]^ “Bulk-related” symptoms such as urine incontinence, stool constipation, and pain in the sacral back area are less typical manifestations of uterine fibroids, resulting from the compression of surrounding organs. They can vary depending on the size and location of the leiomyoma, and were also reported by our patient.^[8,19]^ Ultrasonography is the first-line imaging procedure for suspected leiomyoma because of its high specificity and sensitivity, reaching 99%.^[3]^ Heterogeneous echo-structures are a typical sonographic characterization of uterine fibroids.^[20]^

Some of the known risk factors for the development of uterine fibroids that could have had an impact on the patient’s medical condition were age, time since last birth, and hypertension. The factors that could have had a protective effect in this case were parity and postmenopausal stage.^[1,8]^ In postmenopausal women presenting with symptomatic myomas, hysteroscopy with or without bilateral salpingo-oophorectomy is the most effective treatment. Abdominal, laparoscopic, or vaginal approaches can be considered. Transvaginal and laparoscopic hysterectomies are associated with decreased pain, blood loss, and recovery time compared with transabdominal surgery; however, the vaginal route is limited by the size of the myomatous uterus. Hence, the treatment should be individualized for each patient, and in the described case (due to the size and emergency), abdominal surgery was chosen as the optimal option.^[3,8]^ Moreover, hysterectomy performed with bilateral salpingo-oophorectomy in women older than 45 years has not been associated with a poorer long-term survival rate according to the study by Tuesley et al (2020).^[21]^ Since vaginal hemorrhage could be life-threatening in some cases, acute uterine bleeding unrelated to pregnancy may require urgent or emergent intervention, for which our patient qualified.^[8]^ It is worth noting that uterine fibroids are dependent on the levels of estrogen and progesterone as well as cytokines, growth factors, and chemokines. Together, they contribute to tumor growth by increasing cell proliferation and survival and enhancing extracellular matrix formation.^[2,8]^ Furthermore, in postmenopausal women presenting with a new onset of uterine fibroid bleeding, leiomyosarcoma should be considered, considering that leiomyomas and leiomyosarcomas cannot be reliably distinguished clinically or by any imaging technique.^[8]^

### 3.2. Granulosa cell tumor

GCT is an infrequent, low-grade malignant tumor. This can occur during any period of life. Juvenile GCTs represent only 5% of cases, which appear mostly in pubertal women under 30 years of age. The majority of affected patients are diagnosed between 40 and 70 years, with a median age of 50 years.^[22,23]^ Therefore, the patient in the present report can be classified within an average time range of onset. Fertility, drugs, oral contraceptives, or mutations in breast cancer gene 1 and breast cancer gene 2 have not been proven to have clinical and biological significance in GCT incidence. The patient was multiparous and postmenopausal, but neither menopausal status nor parity was associated with a higher risk.^[13,14]^ The prognosis of GCTs is favorable in most cases.^[24]^ The prognostic factors that have been noted to have important significance are stage, age, tumor size, and extent of surgery, whereas only the stage seems to correlate with recurrence.^[14,25]^ Using the The International Federation of Gynecology and Obstetrics classification, in the described patient’s case, the tumor was classified as stage I due to its isolation to the ovary. The assessed stage is currently the most common stage found across patients suffering from GCT (78–91% of cases).^[10,14]^ Abnormal uterine bleeding and pain are the most common manifestations of GCTs.^[10]^ Most often, the pain is caused by the large size of the tumor, although a more acute onset may be a demonstration of ovarian torsion.^[14]^ In our patient, these symptoms were present before hospitalization. GCTs are unlikely to be the cause of patient’s pain due to their small size. Other symptoms described in the literature include distension mass, menstrual disturbances, abdominal discomfort, increasing abdominal circumference, and weight loss, which were not found in our patient.^[10]^ Finally, GCT may produce both estrogens and androgens or be hormone-inactive; nevertheless, studies of its endocrine function are rarely done, as the diagnosis is often made after the excision of a tumor during surgery. Endogenous estrogen effect is associated with symptoms of the tumor, such as postmenopausal bleeding, a higher incidence of endometrial abnormalities, and breast cancer.^[22]^ In the present case, estrogen levels were not tested because of the need for rapid diagnosis and surgical procedures. Therefore, the possible hormonal impact of GCT on the uterus and its symptoms is unknown. Hysterectomy with bilateral salpingo-oophorectomy, which was performed in this patient’s case, is the standard treatment for early stage GCT in postmenopausal women. However, the patient qualified for this surgical procedure due to other indications, such as the presence of cervical leiomyoma. For stage II to IV GCTs, adjuvant postoperative therapy is recommended. Nevertheless, the benefits of chemotherapy and radiotherapy are still vague because of the lack of randomized trials.^[14,24,26]^ Prolonged surveillance is required, as adult granulosa cell tumors tend to relapse years after the initial diagnosis.^[27]^

### 3.3. Coincidence of cervical leiomyoma and granulosa cell tumors

Estrogen hypersecretion results in the induction or accelerated growth of leiomyomas. Moreover, it can be a cause of postmenopausal bleeding.^[28]^ Thus, it is highly possible that the presence of GCT influenced the development of leiomyoma in our patient at several levels. This could include the massive overgrowth of their cervical leiomyoma (typical cervical leiomyoma tumor’s diameters reach up to 1.0 cm, while in our patient’s case it was 9.04 × 7.81 cm) and their excessive, life-threatening bleeding. The bleeding experienced by the patient is also an atypical symptom, as leiomyoma can rarely cause massive bleeding leading to hypovolemia,^[29]^ which could have been a presumable consequence in the patient’s case.

Hysterectomy with bilateral salpingo-oophorectomy is a typical surgical treatment in postmenopausal women for both of the tumors experienced by this patient. It is worth mentioning that GCT is a rare cause of leiomyoma recurrence, even in post-hysterectomy stumps.^[30]^

As GCT has a tendency for late recurrence and is potentially malignant, a long-term follow-up period of more than 3 decades is necessary. It is recommended that patients undergo pelvic examinations and tumor marker assessments every 3 months (for the first 2 years) and then every 6 months after the third year. Usually, a computer tomography scan of the abdomen and pelvis is performed.^[24]^

## 4. Conclusion

Although it was not possible to measure the estrogen levels in the presented patient’s case, we assume that the coincidence of GCT with leiomyoma could be the reason for its exceptional clinical presentation, based on the cases previously mentioned in the literature. It is probable that the enormous size of the patient’s cervical leiomyoma and severe vaginal bleeding were caused by excessive estrogen secretion by their GCT. Furthermore, the cervical tumor’s location could have led to increased hemorrhaging.

The patient underwent a surgical procedure, which was the standard treatment in postmenopausal women for both of the tumors experienced by this patient. However, in patients of reproductive age who are diagnosed with leiomyoma alone, more conservative surgical management is usually performed. Therefore, in situations where alarming symptoms such as heavy bleeding or extraordinarily large tumor sizes appear with leiomyoma, it is important to remember to search for other possible reasons for the patient’s abnormal condition, including GCTs.

## Acknowledgements

The publication was funded by sources from the Wroclaw Medical University Medical Research Council sources. All authors read and agreed to the published version of the manuscript.

Due to the patient’s age is considered as vulnerable population, we have obtained an informed and written consent. The document was signed directly by the patient who was holding a full legal capacity and did not have a legal guardian. Furthermore, the signatures were collected by both parties after the talk between the patient and the senior author (who is being the attending physician responsible for the patient). Additionally, we carefully wrote the manuscript and prepare the figures in a manner that did not allow identification of the patient.

The study is a case report describing the rare coincidence. It present valuable insight into clinical diagnosis and management, in the case of coexisting particular symptoms and conditions described in the work. Nevertheless, it is essential to highlight the single cage study cannot be directly generalized into a wider population. Lack of the experimental conditions and control groups unable to state common relations between described medical conditions, symptoms and diagnosis.

## Author contributions

**Conceptualization:** Rafal Sozański.

**Data curation:** Rafal Sozański.

**Formal analysis:** Rafal Sozański.

**Funding acquisition:** Rafal Sozański.

**Investigation:** Rafal Sozański.

**Methodology:** Rafal Sozański.

**Project administration:** Rafal Sozański.

**Resources:** Rafal Sozański.

**Software:** Rafal Sozański.

**Supervision:** Rafal Sozański.

**Validation:** Rafal Sozański.

**Visualization:** Rafal Sozański.

**Writing – original draft:** Krzysztof Kotowski, Paulina Małyszczak, Magdalena Towarek, Arkadiusz Jagasyk, Rafal Sozański.

**Writing – review & editing:** Marek Murawski, Rafal Sozański.
